# Caesarean Myomectomy among Patients Undergoing Lower Segment Caesarean Section in a Tertiary Care Center

**DOI:** 10.31729/jnma.6160

**Published:** 2021-02-28

**Authors:** Sapana Amatya Vaidya, Rijuta Jha

**Affiliations:** 1 Department of Obstetrics and Gynecology, Paropakaar Maternity and Women's Hospital Kathmandu, Nepal

**Keywords:** *caesarean section*, *leiomyoma*, *myoma*, *myomectomy*

## Abstract

Pregnancy with uterine myoma increases the risk of abortion, fetal malpresentation, placenta previa, postpartum hemorrhage, hysterectomy and risk to neonate and mother. Caesarian myomectomy is a safe and cost-effective procedure especially when performed by an experienced surgeon only in selected cases. Here, we present our experiences of cesarean myomectomy on ten patients presenting to our center in a period of one year. The most common indications were breech presentation and previous cesarean section. The most common site was anterior, except one which was posterior and the common type is intramural. Despite prophylactic measures, two cases had a postpartum hemorrhage of 2000ml and 700ml, respectively and one even received a blood transfusion. No cases of hysterectomy, neonatal morbidity and mortality were noted in these cases. In our experience, cesarean myomectomy in uterine fibroids has been a safe procedure with limited intraoperative and postoperative complications.

## INTRODUCTION

Uterine myoma is one of the commonest tumors of the reproductive age group. The overall incidence of fibroid uterus is 30-70%.^1^ Incidence of myoma in pregnancy is 0.05-5%.^2^ This may be due to the size of the fibroid that increases during pregnancy and may lead to pain, discomfort, fetal malpresentation, preterm labor, premature rupture of membrane, obstructed labor, abruptio placenta, uterine atony, and PPH.^3,4,7^

The procedure itself causes an increased risk of postoperative morbidity, but new studies suggest that the myomectomy can be carried out during cesarean section in selected patients and if various factors such as uterine contractility, anatomic localization, number and diameter of myomas, and the presence of vascular structures are taken into account.^5-9^

## CASE ANALYSIS

The age of the patients varied from 23 to 34 years. Among ten patients, 60% were primipara and 40% multipara. All were term pregnancy except one Preterm at 33 weeks and six days of pregnancy. Of the total of ten patients, six were for elective lower segment cesarean section (LSCS) and four emergency LSCS. Regarding presentation, 4 out of ten were breech presentations. Almost all myomas except one were on the anterior surface of the uterus. The smallest size of the myoma was 2x3 cm, and the largest was 14x12 cm. Both were an anterior wall. Six out of ten were intramural, including the posterior one. All cases had a single myoma (Table 1).

**Table 1:** Clinical profile of patients undergoing cesarean myomectomy.

S. N	Age	Prim/Multi para	Weeks of gestation	Indication of LSCS	Emergency/Elective LSCS	Presentation of the baby	Site/number of myoma	Size/type of Myoma
1	23	Primipara	37+6	Breech with fibroid uterus	Elective	Breech	Anterior lower segment 'one	3x3cm Subserosal
2	32	Primipara	33+6	PPROM with primary infertility	Emergency	cephalic	Anterior lower segment Single	2x3cm Subserosal
3	34	G2Po+1	39	Breech with fibroid uterus	Elective	Breech	Anterior upper segment one	4x5cm Intramural
4	28	G2P2L2	38weeks	Refused VBAC with previous CS	Elective	Cephalic	Anterior lower segment Single	3x3cm Intramural
5	22	Primipara	37+5	Breech with myoma	Elective	Breech	Anterior surface more towards the fundal region Single	14x12cm Subserous
6	33	G3P1L1	39+5	Prev LSCS refused VBAC	Elective	Cephalic	Anterior lateral Upper segment, single	5x6cm Intramural
7	24	Primipara	39+6	Breech with fibroid GTH in LPOL	Emergency	Breech	Posterior lower region One in no	10x7cm Intramural
8	33	Primipara	37+6	Primary infertility (IUI conception) with Knee Arthrodesis and fibroid uterus in LPOL	Emergency	Cephalic	Anterior near to lower segment Single	5x6cm Subserous
9	28	G3P1+1L1	39+	Prev LSCS with CPD with fibroid uterus	Elective	Cephalic	Anterior lower segment One in no	6x6cm Intramural
10	26	Primipara	39+	Non reassuring CTG	Emergency	Cephalic	Anterior wall One,	5x5cm Intramural

Blood loss ranged from 150 ml to 2000 ml, with two cases of postpartum hemorrhage. Only one patient with a blood loss of 2000 ml has received a blood transfusion. None of the patients had undergone a hysterectomy. In one case, prophylactic measures had blood loss of 2000 ml, received uterine tourniquet, injection Tranexamic acid (1gm), Tab Misoprostol (800 mcg), and Inj Carboprost. Another PPH case with 700 ml loss received temporary bilateral infundibulopelvic ligament clamping along with Tranexamic acid (1gm) and Tab Misoprostol (800 mcg). Five cases received both Injection Tranexamic acid (1gm) and tab Misoprostol (800 mcg) and the rest three cases either received injection Tranexamic (1gm) or tab misoprostol (800 mcg). The total duration of stay was 3-5 days (Table 2).

**Table 2:** The outcome of patients undergoing cesarean myomectomy.

S.N	Blood loss	Complication			Prophylactic measures			
		PPH	The need for blood transfusion	Hysterectomy	uterine tourniquet,	Bilateral uterine artery ligation	Bilateral infundibulo Pelvic Ligament Temporary Clamping	Uterotonic as Injection Tranexamic acid, Misoprostol, Carbopost, Methergine
1	200 ml	No	No	No	No	No	No	Tranexamic acid, Tab. Misoprostol
2	400 ml							Tranexamic acid Tab misoprostol
3	150 ml							Tab misoprostol
4	250 ml							Tranexamic acid
5	2000 ml	Yes	Yes		Yes			Tab tranexamic acid Tab misoprostrol Inj Carboprost
6	700 ml	Yes					Yes	Inj Tranexamic acid Tab misoprostrol
7	400 ml						Yes	Tab misoprostol
8	300 ml							Inj Tranexamic acid Tab misoprostol
9	400 ml							Inj Tranexamic acid Tab misoprostol
10	400 ml							Inj Tranexamic acid Tab misoprostol

## CASE I

Cesarean myomectomy with minimal blood loss was done in a 23-year-old primipara at 37+6 weeks with breech presentation and fibroid uterus. A subserosal myoma of size 3cm x 3cm was found in the lower segment of the uterus

## CASE II

She was a 32-year-old primipara at 33+6 weeks of gestation. PPROM with primary infertility was the indication for emergency LSCS. During surgery, she had a subserosal myoma of size 2cm x 3cm. The smallest in size of the myomas among ten cases in the lower segment of the uterus. Out of ten, this case is the only case of preterm premature rupture of the membrane with infertility.

## CASE III

A 34-year-old G2P1 presented at 39 weeks of gestation underwent elective LSCS presented for breech. An intramural uterine fibroid of size 4x5cm was during the surgery and a cesarean myomectomy was performed. Blood loss was minimal, about 150ml, which is the least in the case series.

## CASE IV

A 28-year-old female with a history of previous cesarean section (G2P2L2) presented at 38 weeks of gestation underwent elective LSCS for refused VABC. An intraoperative anterior lower segment intramural fibroid of 3cm x 3cm was discovered and underwent a cesarean myomectomy. She received only an injection of Tranexamic acid as a prophylactic measure with blood loss of only 250 ml.

## CASE V

A 22-year-old primipara underwent elective lower segment cesarean section with myomectomy 37+5 weeks of gestation for breech presentation and uterine fibroid. A single subserosal fibroid of 14 cm x 12 cm was present on the anterior surface more towards the fundal region and the largest in the series. Postpartum hemorrhage of 2000ml occurred despite prophylactic measures with the application of a uterine tourniquet and uterotonic and inj Tranexamic acid. This is the only case that received a blood transfusion.

## CASE VI

The elective cesarean section was done for the previous cesarean section with refused VABC is a 33-year-old G3P1L1 presented at 39+5 weeks of gestation. A 5cm x 6cm intramural myoma was present over the anterior lateral portion of the upper segment of the uterus, for which myomectomy was done. Despite a PPH of 700 ml, the patient did not receive a blood transfusion. The patient received prophylactic bilateral temporary infundibulopelvic ligament clamping, inj Tranexamic acid and tab misoprostrol 800 μgm.

## CASE VII

An emergency LSCS with myomectomy was performed in a 24-year-old primipara who presented at 39+6 weeks for breech with fibroid and GTH in LPOL. A single 10 cm x 7 cm intramural myoma was present in the posterior lower region. This is the only case with posterior myoma and with associated medical problems. No PPH and no maternal and neonatal morbidity noted

## CASE VIII

A 33-year-old primipara at 37+6 weeks underwent emergency cesarean section with myomectomy for primary infertility (IUI conception) with fibroid uterus and knee arthrodesis in LPOL. The subserosal fibroid was 5 cm x 6 cm in size present in the uterus's anterior portion near the lower segment. Total blood loss was 300 ml, and both Tranexamic injection acid and tab Misoprostrol were given as prophylactic measures.

## CASE IX

A 28-year-old G3P1+1L1 at 39+ weeks of gestation with a history of previous cesarean section with cephalopelvic disproportion and fibroid uterus underwent elective cesarean myomectomy. Removed intramural myoma of 6x6 cm in the anterior lower segment and was uneventful.

## CASE X

A 26-year-old primipara at 39+ weeks of gestation underwent emergency LSCS following non-reassuring CTG. Intramural fibroid of 5 cm x 5 cm size was seen in the anterior wall of the uterus. For this caesarean myomectomy was done with no PPH and no morbidity to mother and baby.

## DISCUSSION

Fibroids can affect pregnancy outcomes as they can lead to an increased risk of spontaneous abortion, fetal malpresentation, placenta previa, preterm birth, cesarean section, postpartum hemorrhage etc.^10^ Most pregnancies associated with fibroid remains uneventful, but one out of ten pregnant women with fibroid develop a complication.^3^ In our case, two out of ten total cases developed PPH and one received a blood transfusion.

Combining myomectomy with cesarean section has been discouraged previously, mainly due to risk of intractable hemorrhage, failure to obliterate the cavity, and eventually landing into a hysterectomy.^8^ Despite the significant progress in medical and non-surgical myoma management, cesarean myomectomy is still a controversial issue.^8,10^ However, newer evidence suggests cesarean myomectomy has become a safe and cost-effective procedure if selected carefully and performed by experienced obstetricians

It benefits two operations in one (CS and myomectomy), thus averting both the risks and costs of reoperation.^3^ However, its application remains a dilemma due to the associated fear of uncontrollable hemorrhage resulting in cesarean myomectomy (Reddi Rani P et al.). About 40.9% of patients who had only CS while also having myoma required repeat surgery during follow-up of 6-38 months for symptomatic myoma.^4^

There are advantages of myomectomy during cesarean section over interval myomectomy. An incision on the uterus is generally smaller as the uterus to tumor ratio is smaller than in non-pregnant. Myomectomy itself is technically easier to perform due to easier identification of the cleavage plane.^10^ The pregnant uterus' elasticity enables the effortless placement of stitches, while uterine contractions and physiological involution in the puerperium further reduce hemorrhage.^10,11^ Myomectomy during cesarean section benefits two operations in one thus obviating risks and cost of reoperation.^12^

In a study done by Kant et al. in 9 cesarean sections, the most common indication was fetal distress, followed by cephalopelvic disproportion, fetal malposition, and the previous two cesarean sections.^12^ However, in our study, the most common indications were breech with fibroid uterus and previous cesarean section refusing VBAC. Michalas et al. reported a case where eight fibroids in the lower part of the uterus were removed using a cesarean section without complications.^12^ In our study, 50% of the surgeries were performed on the fibroids in the lower section, and all were single.

Kaymak et al.compared 40 patients who underwent cesarean myomectomy with 80 patients who had myoma and undergone Caesarean section alone. The fibroids' mean size was 8.1 cm in the cesarean myomectomy group to 5.7 cm in the cesarean group only. There was no significant difference in hemorrhage incidence, 12.5% to 11.3% in the control group.^2^ In our study, the incidence of hemorrhage is 20 % which is more than that in the study.

In a series of nine, Exacoustos et al. reported three cesarean hysterectomies,^5^ while Pattanaik et al. reported one subtotal hysterectomy out of 23 CM.^6^ However, in our study, no hysterectomy was performed.

In Our study of the total of ten cases, the age range was from 23 to 34 years which is a little bit different to 23-48 years in a study conducted by Ashley S Roman and Khalil MA Tabsh.^1^ This may be due to the high number of cases in the compared study.

Senturk et al., in their retrospective study with 361 patients, compared cesarean section with and without myomectomy where they found myomectomy did not increase complications or transfusion rates.^13^ The authors concluded that the procedure appeared to be safe. We have a similar finding from our study where only two patients had blood loss, of which one received a blood transfusion. Nonetheless, the procedure is associated with an increase in the risk of intraoperative hemorrhage.^14^

Cesarean myomectomy can be performed safely if the selection of case is appropriate with appropriate prophylactic measures and experienced hands. This really helps prevent the risk of two operations and eventually a cost-effective, safe procedure.
